# Risk Factors Associated With Inadequate Brain Relaxation in Craniotomy for Surgery of Supratentorial Tumors

**DOI:** 10.7759/cureus.25544

**Published:** 2022-05-31

**Authors:** Natalia Pérez de Arriba, Aida Antuña Ramos, Vanesa Martin Fernandez, Maria del Carmen Rodriguez Sanchez, Jose Ricardo Gonzalez Alarcon, Marco Antonio Alvarez Vega

**Affiliations:** 1 Anesthesiology and Reanimation, University Central Hospital of Asturias (HUCA), Oviedo, ESP; 2 Neurosurgery, University Central Hospital of Asturias (HUCA), Oviedo, ESP; 3 Cellular Morphology and Biology, Faculty of Medicine and Health Sciences, University of Oviedo, Oviedo, ESP

**Keywords:** brain relaxation, dexamethasone, mannitol, cerebral tumors, cerebral swelling

## Abstract

Introduction: Cerebral swelling often occurs during craniotomy for cerebral tumors. Poor brain relaxation can increase the risk of cerebral ischemia, possibly worsening the outcome. The surgical team should identify any risk factors that could cause perioperative brain swelling and decide which therapies are indicated for improving it. The present investigation aimed to elucidate the risk factors associated with brain swelling during elective craniotomy for supratentorial brain tumors.

Methods: This prospective, nonrandomized, observational study included 52 patients scheduled for elective supratentorial tumor surgery. The degree of brain relaxation was classified upon the opening of the dura according to a four-point scale (brain relaxation score: 1, perfectly relaxed; 2, satisfactorily relaxed; 3, firm brain; and 4, bulging brain). Moreover, hemodynamic and respiratory parameters, arterial blood gas, and plasma osmolality were recorded after the removal of the bone flap.

Results: This study showed that the use of preoperative dexamethasone was associated with a brain relaxation score of ≤2 (p = 0.005). The median midline shift of 6 (3-0) mm and median hemoglobin level of >13 g/dL were associated with a brain relaxation score of ≥3 (p = 0.02 and p = 0.01, respectively). The dosage of mannitol (0.25 g/kg^ ^versus 0.5 g/kg), physical status, intraoperative position, tumor diameter and volume, peritumoral edema and mass effect, World Health Organization (WHO) grading, mean arterial pressure, PaCO_2_, osmolality, and core temperature were not identified as risk factors associated with poor relaxation.

Conclusion: The use of preoperative dexamethasone was associated with improved brain relaxation, whereas the presence of a preoperative midline shift and a higher level of hemoglobin were associated with poor brain relaxation.

## Introduction

Brain relaxation is the relationship between the volume of the intracranial contents (the brain, cerebrospinal fluid, and blood) and the capacity of the intracranial space when the cranium and dura are opened by the neurosurgeon. Optimal brain relaxation improves the operating conditions, minimizes the severity of retraction injury and the risk of cerebral ischemia, and offers the potential for an improved postoperative outcome [1].

The surgical team should identify risk factors that could lead to perioperative brain swelling and decide with the neurosurgeon which therapy is indicated before opening. Various parameters may affect the success of brain relaxation in craniotomy performed for the management of supratentorial tumors, such as the type and dosage of osmotherapy, the presence of midline shift, peritumoral edema, histology (e.g., glioblastoma multiforme or metastasis), and a subdural pressure of more than 13 mmHg [1-5].

Therefore, the present investigation aimed to elucidate the risk factors associated with brain swelling during elective craniotomy for supratentorial brain tumors. Risk factors included patient characteristics (age, clinical status, and intraoperative position), neuroimaging findings (tumor diameter, volume, location, midline shift, peritumoral edema, and mass effect), preoperative use of dexamethasone, the intraoperative dose of mannitol, physiological values (mean arterial pressure [MAP] and core temperature), analytical data (hemoglobin [Hb], osmolality, and PaCO_2_), and histopathological features.

## Materials and methods

This single-center, prospective, nonrandomized, observational study was approved by the Ethical Committee of Hospital Universitario Central de Asturias (HUCA) (Oviedo, Spain) (Ref. no.: 183/17) on August 22, 2017, and by the Spanish Agency of Medicines and Health Products (Ref. no.: NPA-MAN-2017-01) on December 4, 2017. Written informed consent was obtained from all patients before participation. The trial was conducted in accordance with the ethical principles outlined in the Declaration of Helsinki, revised in 2000.

Subjects scheduled to undergo craniotomy for supratentorial tumors were included in this study. All the surgeries were approved by the members of the Tumor Committee of HUCA. The exclusion criteria were a preoperative Glasgow Coma Scale (GCS) score of 13 points or less, intraoperative use of 20% mannitol at more than 0.5 g/kg, intraoperative use of hypertonic saline and/or furosemide, treatment with a hyperosmotic agent (mannitol or hypertonic saline) in the previous 24 hours, the presence of a preoperative disturbance of water or sodium metabolism (diabetes insipidus, cerebral salt wasting syndrome, or syndrome of inappropriate antidiuretic hormone secretion), awake craniotomy, obstructive hydrocephalus, and ventricular or lumbar drainage. In Spain, the approved doses of 20% mannitol for reducing intracranial pressure (ICP) are 0.25-0.5 g/kg [1,6], so only patients who received dosages of 0.25 g/kg or 0.5 g/kg were assessed for the study.

Anesthetic procedure

After arriving in the operating room, the patient was monitored using noninvasive blood pressure, pulse oximetry, and five-channel electrocardiography. All patients received 12 mg of dexamethasone intravenously before skin incision, according to our standard clinical practice. No other premedication was administered. General anesthesia was induced and maintained with a target-controlled infusion (TCI) of propofol (Schnider pharmacokinetic model, initial effect-site concentration [Ce] of 2 µg/mL) to achieve bispectral index values between 40 and 60 (BIS®, Covidien LCC, Mansfield, MA, USA) and a TCI of remifentanil (Minto pharmacokinetic model, initial Ce of 2-3 ng/mL).

Tracheal intubation was facilitated with rocuronium (0.6-1.2 mg/kg). Muscle relaxants were used as needed to maintain a single twitch upon train-of-four stimulation. Mechanical ventilation was conducted with oxygen (fraction of inspired O_2_ 40%-50%)-air mixture, and end-tidal CO_2_ was continually monitored. After orotracheal intubation, a radial or humeral catheter and urinary catheter were inserted. Zero was positioned at the Monro foramen level. The core temperature was measured using an esophageal probe. The patients’ heads were positioned carefully, avoiding extreme neck flexion or over-rotation to maintain cerebral venous drainage.

Using an infusion pump, 20% mannitol (1,110 mOsm/L) was administered at the skin incision through a peripheral line. The dose of mannitol was chosen by the attending anesthesiologist, and the surgeon was blinded to the amount of mannitol given to the patient. A balanced crystalloid solution (Plasmalyte, Baxter S.L., Valencia, Spain) was infused as needed to replace fluids lost in the urine output.

The brain relaxation was scored by the most experienced neurosurgeon upon the opening of the dura using a four-point scale (brain relaxation score [BRS]), with one point awarded for a perfectly relaxed brain, two points awarded for a satisfactorily relaxed brain, three points awarded for a firm brain, and four points awarded for a bulging brain [7]. Hb and blood gases (ABL90 Flex, Radiometer Medical ApS, Denmark), plasma osmolality (Osmo Station OM-6060, Arkray Factory Inc., Kyoto, Japan), MAP, and core temperature were recorded immediately before opening the dura mater.

Tumor features

The tumor’s largest diameter and volume, location, midline shift, mass effect, and peritumoral edema on preoperative computerized axial tomography or magnetic resonance imaging scans were evaluated by an independent radiologist. Mass effect was assessed according to the Gordon-Firing scale, where zero points indicated no mass effect, one point indicated compression of the convexity sulci, two points indicated compression of the ipsilateral ventricle, three points indicated midline shift, and four points indicated contralateral ventricular dilatation [8]. The Steinhoff classification scheme was used to assess peritumoral edema as follows: zero points indicated no signs of edema, one point indicated peritumoral edema limited to 2 cm, two points indicated peritumoral edema limited to half of the hemisphere, and three points indicated peritumoral edema in more than half of the hemisphere [9]. The histopathological diagnosis, defined according to World Health Organization (WHO) grading scheme, was obtained from the postoperative neuropathological report [10].

Statistical analysis

To analyze the effect of 20% mannitol on BRS, we performed a power calculation to determine the ideal sample size before the initiation of the study. On the basis of previous studies [11,12] and the assumption that a difference of one unit on BRS from 1 to 4 in brain relaxation is clinically relevant, α was set to 0.05 and β to 0.9, and the sample size of 44 patients was calculated. Considering a loss ratio of 15%, this calculation produced a sample size of 52 subjects.

Categorical variables are expressed as numbers of patients and percentages (%), and quantitative variables are expressed as mean ± standard deviations (SD) or medians and interquartile ranges (IQs) (25th percentile to 75th percentile). The chi-squared test or Fisher’s exact test was used for categorical data analysis. The student’s t-test or Wilcoxon’s test was used for quantitative data analysis after testing for normality with the Kolmogorov-Smirnov test. A p-value of less than 0.05 was considered statistically significant. Statistical analyses were performed with R statistical software, version 3.4.4 (http://www.r-project.org/; the R Foundation for Statistical Computing, Vienna, Austria).

## Results

The data were collected between September 2017 and December 2020. A total of 325 patients were considered for the study. Of this number, 273 patients did not meet the inclusion criteria (lumbar drainage: 28, awake craniotomy: 12, no intraoperative use of mannitol: 56, use of 20% mannitol > 0.5 g/kg: 133, use of hypertonic saline: 12, and use of furosemide: 3), or refused to participate in the study (16). Fifty-two patients completed the study and were included in the data final analysis. The preoperative characteristics of the patients and tumor features are summarized in Tables 1, 2, respectively.

**Table 1:** Patients' characteristics GCS: Glasgow Coma Scale; ASA: American Society of Anesthesiologist physical status.

Patient characteristics	n	%
Age (years) < 65	31	59.6
Age (years) ≥ 65	21	40.4
Male	23	44.2
Female	29	55.8
Preoperative GCS ≥ 13	52	100
ASA physical status ≤ II	46	88.4
ASA physical status ≥ III	6	11.6
Preoperative use of dexamethasone	28	53.8
No preoperative use of dexamethasone	24	46.2
Intraoperative position: Supine + Lateral	45	88,5
Intraoperative position: Prone + Three-quarters	7	13,5
Mannitol group: 0.25 g/kg	26	50
Mannitol group: 0.5 g/kg	26	50

**Table 2:** Tumor features WHO: World Health Organization.

Tumor characteristics	n	%
Days between radiological studies and surgery ≤ 7	4	7.69
Days between radiological studies and surgery > 7	48	92.3
Tumor largest diameter (cm) < 3	12	23
Tumor largest diameter (cm) ≥ 3	40	77
Location: 1 lobe	41	78.8
Location ≥ 2 lobes	11	21.1
Midline shift	22	42.3
No midline shift	30	57.7
Mass effect (Gordon-Firing Scale): 0/I/II	38	73
Mass effect (Gordon-Firing Scale): III/IV	14	26.9
Peritumoral edema (Steinhoff classification): 0/I	34	65.3
Peritumoral edema (Steinhoff classification): II/III	18	34.6
WHO grading: 1/2	37	71.2
WHO grading: 3/4	15	28.8

The association between BRS and risk factors of brain swelling is presented in Table 3, and the physiological variables and laboratory data are presented in Table 4.

**Table 3:** Association between brain relaxation and risk factors of brain swelling *p < 0.05. BRS: Brain relaxation score; ASA: American Society of Anesthesiologists; WHO: World Health Organization.

Risk Factors	BRS ≤ 2	BRS ≥ 3	p-value
Number of patients	44	8	
% Age (years) < 65	63.6	37.5	0.24
% Age (years) ≥ 65	36.3	62.5	0.24
% ASA physical status ≤ II	86.3	100	0.57
% ASA physical status ≥ III	13.63	0	0.57
% Intraoperative position: Supine + Lateral	90.9	62.5	0.06
% Intraoperative position: Prone + Three-quarters	9.09	37.5	0.06
% Preoperative use of dexamethasone	45.5	100	0.005^*^
% No preoperative use of dexamethasone	54.5	0	
% Mannitol group: 0.25 g/kg	47.7	62.5	0.73
% Mannitol group: 0.5 g/kg	52.7	37.5	0.73
% Tumor largest diameter < 3 cm	25	12.5	0.66
% Tumor largest diameter ≥ 3 cm	75	87.5	0.66
% Location: 1 lobe	75	100	0.51
% Location ≥2 lobes	25	0	0.51
% Midline shift present	36.3	75	0.05^*^
% No midline shift present	63.6	25	0.05^*^
% Mass effect (Gordon-Firing scale): 0, I, II	75	62.5	0.48
% Mass effect (Gordon-Firing scale): III, IV	25	37.5	0.48
% Peritumoral edema (Steinhoff classification): 0, I	70.45	37.5	0.14
% Peritumoral edema (Steinhoff classification): II, III	29.54	62.5	0.14
% WHO grading, low grade (1 + 2)	72.7	62.5	0.67
% WHO grading, high grade (3 + 4)	27.2	37.5	0.67

**Table 4:** Association between brain relaxation and risk factors of brain swelling: variables and laboratory data *p < 0.05. BRS: Brain relaxation score; MAP: Mean arterial pressure; SD: Standard deviation.

Risk Factors	BRS ≤ 2	BRS ≥ 3	p-value
Number of patients	44	8	
MAP (mmHg), mean ± SD	80.5 ± 16.6	80.8 ± 10.4	0.93
PaCO_2_ (mmHg), median ± SD	36.8 ± 2.3	39.6 ± 3.1	0.19
Hemoglobin (g/dl), mean ± SD	12.2 ± 1	13.1 ± 1	0.01*
Osmolality (mOsm/kg), mean ± SD	293.6 ± 5.5	295 ± 5.8	0.54
Core temperature (ºC), mean ± SD	35.5 ± 0.5	35.4 ± 0.4	0.54

Age, American Society of Anesthesiologists (ASA) physical status, intraoperative position, tumor diameter and volume, location, mass effect, peritumoral edema, and WHO grading were not associated with poor brain relaxation. MAP, PaCO_2_, osmolality, and core temperature did not differ between patients with a BRS ≤ 2 and those with a BRS ≥ 3. Regarding the dose of mannitol, 26 patients received 20% mannitol 0.25 g/kg (group M 0.25) and 26 patients received 20% mannitol 0.5 g/kg (group M 0.5) (Tables 5, 6).

**Table 5:** Comparison between group 0.25 g/kg and group 0.5 g/kg 20% mannitol: patient and tumor characteristics *p < 0.05. M: Mannitol; ASA: American Society of Anesthesiologists; GCS: Glasgow Coma Scale; WHO: World Health Organization.

Risk Factors	M 0.25	M 0.5	p-value
Number of patients	26	26	
% Age (year) < 65	42.3	76.92	0.02*
% Age (year) ≥ 65	57,6	23.7	0.02*
% Male	46.15	42.3	1.00
% Female	53.8	57.6	1.00
% ASA physical status ≤ II	84.6	92.3	0.66
% ASA physical status ≥ III	15.38	7.69	0.66
% Preoperative GCS ≥ 13	100	100	1.00
% Intraoperative position: Supine + Lateral	80.7	92.3	0.41
% Intraoperative position: Prone + Three-quarters	19.23	7.69	0.41
% Preoperative use of dexamethasone	57.6	50	0.78
% No preoperative use of dexamethasone	42.3	50	0.78
% Tumor largest diameter < 3 cm	23.07	23.07	1
% Tumor largest diameter ≥ 3 cm	76.9	76.9	1
% Location: 1 lobe	76.9	80.79	1.00
% Location ≥2 lobes	23,07	19.23	1.00
% Midline shift present	38.46	46.15	0.33
% No midline shift present	61.53	53.86	0.33
% Mass effect (Gordon-Firing scale): 0, I, II	73.07	73.07	0.86
% Mass effect (Gordon-Firing scale): III, IV	26.9	43.75	0.86
% Peritumoral edema (Steinhoff classification): 0, I	65.38	65.38	0.58
% Peritumoral edema (Steinhoff classification): II, III	34.61	34.61	0.58
% WHO grading, low grade (1 + 2)	65.38	76.09	0.54
% WHO grading, high grade (3 + 4)	34.6	23.07	0.54

**Table 6:** Comparison between group 0.25 g/kg and group 0.5 g/kg 20% mannitol: physiological variables and laboratory data *p < 0.05. M: Mannitol; MAP: Mean arterial pressure; SD: Standard deviation.

Risk Factors	M 0.25	M 0.5	p-value
Number of patients	26	26	
MAP (mmHg), mean ± SD	78 ± 16.6	81 ± 10.4	0.93
PaCO_2_ (mmHg), mean ± SD	37.51 ± 2.8	36.94 ± 4.6	0.19
Hemoglobin (g/dl), mean ± SD	12.2 ± 1.4	13.1 ± 1.2	0.01*
Osmolality (mOsm/kg), mean ± SD	293.6 ± 5.6	295 ± 5.4	0.54
Core temperature (ºC), mean ± SD	35.4 ± 0.5	35.6 ± 0.4	0.54

No statistically significant differences were observed between the groups regarding sex, weight, ASA physical status, preoperative GCS, preoperative use of dexamethasone, or intraoperative position. However, a significant difference was found related to age (p = 0.029). Patients aged 65 years or older were more frequently found in group M 0.25, and those younger than 65 years were more frequently found in group M 0.5. No other significant differences were observed regarding the tumor volume, largest diameter, midline shift, mass effect, location, peritumoral edema, WHO grade, or histopathological diagnosis. Hemodynamic variables, laboratory data, PaCO_2_, and core temperature were also similar between the groups (p > 0.05).

Furthermore, no significant difference was noted in osmolality between the groups (293.5 ± 6 mOsm/kg in group M 0.25 and 293.9 ± 5.4 mOsm/kg in group M 0.5; p = 0.84). The effect of 20% mannitol on BRS did not differ between the doses. Overall, 80.7% of the patients in group M 0.25 and 88.4% in group M 0.5 had a BRS of ≤2, whereas 19.2% of patients in group M 0.25 and 11.5% of patients in group M 0.5 presented a BRS of ≥3 (p = 0.73). The median (IQR) of BRS was 2 (1-2) points in both groups. In a sub-analysis of each group, no significant differences were observed regarding intragroup BRS between ages <65 and ≥65 years (M 0.25, p = 0.053; M 0.5, p = 1) (data not included in the table).

Furthermore, preoperative use of dexamethasone was identified as a risk factor for a BRS ≤2 (p = 0.005). However, the presence of midline shift and a higher level of Hb were associated with a BRS ≥ 3. The median (IQR) midline shift in the context of a BRS ≤2 was 0 (0-4.17) mm, and for a BRS ≥3, it was 6 (3-10) mm (p = 0.02). Hb (mean ± SD) was 12 ± 1 g/dL in the context of a BRS ≤ 2 and 13.1 ± 1 g/dL for a BRS ≥ 3 (p = 0.01). The cutoff point for Hb and the midline shift (according to the Youden index) at which patients began to show a BRS ≥ 3 were 13 g/dL and 4 mm, respectively.

## Discussion

Comparison of 0.25 g/kg and 0.5 g/kg 20% mannitol dosages

Mannitol is a commonly administered hyperosmolar agent with a variety of clinical indications. It was introduced to patient care over 50 years ago and is claimed to deliver clinical benefits by reducing ICP and enhancing operative conditions for craniotomy [13,14]. However, the evidence supporting its use is scarce, and the heterogeneity in the dosages used makes it difficult to establish an optimal treatment regimen [15,16].

The clinical utility in patients with disrupted blood-brain barriers (BBBs) is debatable, and the impact on the neurological outcomes and mortality remains unknown. Furthermore, it can cause serious complications, particularly related to cardiovascular changes, electrolyte disturbances, and renal damage in patients with predisposing factors [13,14].

Our study demonstrated that BRS scores were similar between the two dosages of 20% mannitol. This finding is in agreement with the findings of other authors. A randomized study evaluated the effect of a dosage of 150 mL of 20% mannitol (approximately 0.5 g/kg) on the degree of brain relaxation. The patients were scheduled for a supratentorial brain tumor surgery, and the results showed that nearly 97% of the patients displayed a “soft or adequate” brain relaxation outcome using a three-point scale [16]. Additionally, a prospective, randomized clinical trial including 25 patients undergoing supratentorial craniotomy for various brain pathologies (including 20 patients with tumors) showed that the use of 20% mannitol at 0.5 g/kg resulted in a satisfactory degree of brain relaxation when the brain bulk was evaluated as satisfactory or unsatisfactory [17].

More recently, Seo et al. compared the effects of four dosages of 20% mannitol (0.25, 0.5, 1.0, and 1.5 g/kg) on brain bulk for supratentorial brain tumor management according to a four-point scale (1 = bulging, 2 = firm, 3 = adequate, and 4 = perfectly relaxed). The incidence of satisfactory brain relaxation (≥3 points) was significantly higher with dosages of 1.0 and 1.5 g/kg than with 0.25 g/kg, but this was not the case with a dosage of 0.5 g/kg. The proportions of satisfactory brain relaxation were 32.3% and 51.6% in the groups receiving 0.25 and 0.5 g/kg of 20% mannitol, respectively [18].

One of the principal mechanisms of action of mannitol is the promotion of a reduction in the volume of cerebral cells by creating an osmotic gradient between the interstitial and intravascular compartments [13]. Research in animals has shown a significant increase in ICP and cortical water content after a reduction in osmolality of 13 mOsm/kg [19], and it has also been suggested that a significant correlation exists between BRS and serum osmolality after mannitol administration (correlation coefficient: 1.43) [18]. Thus, the lack of a difference in BRS in our study may be explained by the absence of a significant difference in osmolality between the groups.

Preoperative use of dexamethasone

No previous trials have examined the effect of dexamethasone on brain relaxation, but our results revealed a better BRS with the preoperative use of dexamethasone. This may be explained by the pathophysiology of tumor-related brain edema and the effects that steroids have on it.

Edema associated with brain tumors is typically considered to be vasogenic, and it contributes significantly to morbidity. This edema results from the disruption of the BBB, allowing protein-rich fluid to accumulate in the extracellular space [20]. Previous trials have shown that the administration of steroids one to two days before an elective surgical procedure has the potential to reduce edema formation by reducing the permeability of tumor capillaries [21,22]. Moreover, steroid therapy had a beneficial effect on the intracranial volume-pressure response, which is an expression of brain elastance and is equivalent to inverse compliance. This could partly explain why a patient’s neurological status may improve following steroid administration, even when there is a little obvious effect on ICP itself [23].

Midline shift

In this study, we showed that the presence of a midline shift ≥ 4 mm was associated with a BRS ≥ 3. This finding is in agreement with those of other authors, but the threshold value for midline shift from which patients would begin to show a worse relaxation differs among them. Bedford and others revealed that patients with small tumors (<3 cm in diameter) and no evidence of edema, midline shift, or ventricular effacement were never found to develop elevated ICP3.

In another study with 692 patients, subdural pressure of more than 13 mmHg (odds ratio [OR]: 1.9), presence of midline shift (5.4 ± 5.8 mm, OR: 1.6), and histopathological diagnosis of glioblastoma multiforme (OR: 2.1) and metastasis (OR: 2.9) significantly predicted the risk of cerebral swelling after opening the dura in patients with supratentorial cerebral tumors [4]. Quentin et al. compared two doses of 20% mannitol (0.7 and 1.4 g/kg) during elective supratentorial brain tumor surgery (40 patients per group); taking into consideration the effect of midline shift (defined as a deviation of more than 1 mm), they showed that the odds of a one-level improvement in relaxation score (on a four-point scale) in patients who received the higher dose of mannitol was 2.5 times higher than the OR for the low dose of mannitol. An OR of 0.29 indicates that the presence of midline shift is associated with a higher probability of less-favorable relaxation scores [2]. In another study with 60 patients, the effects of hypertonic fluids (3 mL/kg of 20% mannitol or 3% hypertonic saline) on brain relaxation during elective supratentorial craniotomy for diverse neuropathology showed a worse response in patients with midline shift (mm of midline shift not recorded) (37% versus 8%, respectively; OR: 6.6) [8].

Hemoglobin

Our results showed that a higher level of Hb is associated with poor brain relaxation. No studies have explored the association between Hb and brain bulk, although some studies have assessed the influence of blood viscosity on various brain pathologies. In animal models, Muizelaar et al. demonstrated the existence of “viscosity autoregulation,” which is mediated through blood viscosity. In areas of the brain presumed to be normal, cerebral blood flow (CBF) is fairly constant despite the changes in blood pressure or blood viscosity because brain vessel constriction occurs with decreased viscosity and vasodilation occurs with increased viscosity. Therefore, mannitol tends to enhance CBF by decreasing blood viscosity; however, in response to this hyperemia, the cerebral vessels constrict to keep the CBF relatively constant by decreasing the cerebral blood volume with an ensuing decrease in ICP. This autoregulation is closely related to pressure autoregulation, probably through the same mechanism [24,25].

In the clinical setting, increased viscosity in acute brain injury likely enhances microcirculatory failure and may contribute to the extent of cerebral ischemia [26]. In patients with aneurysmal subarachnoid hemorrhage treated surgically, an inverse correlation exists between daily postoperative fluctuations in blood viscosity and the level of consciousness [27]. Whole-blood viscosity is a difficult parameter to measure [28], but the main determinant of blood viscosity is the hematocrit level [29], and hematocrit is typically proportional to Hb in normovolemic and nonanemic patients. Thus, we hypothesize that Hb could be used as an indirect measure of blood viscosity. This is a rare study to identify Hb as a risk factor associated with poor brain relaxation. Therefore, further research is needed to study the effect of viscosity, Hb, and hematocrit on brain relaxation.

Limitations of the study

Our study has several limitations. The four-point scale used in this work to assess brain relaxation is subjective and observer-dependent, but it has been widely used by other authors [2,4,7,8,11,12,15] to assess brain relaxation. This scale remains a major method because surgical conditions are evaluated by attending neurosurgeon, and some decisions, such as increasing the dimensions of craniotomy, initiating hyperventilation, opting for a partial resection, and raising the pressure of brain retractors, are typically based on this evaluation. In this study, ICP was not measured to avoid the risk derived from the insertion of a catheter; additionally, ICP is rarely monitored at our institution during intracranial tumor surgery and is thus seldom used to make intraoperative clinical decisions regarding brain relaxation.

## Conclusions

This study described the perioperative factors that may affect brain relaxation, such as patient features, preoperative treatments, neuroimaging findings, and intraoperative management. The results showed that the use of preoperative dexamethasone was associated with improved brain relaxation, whereas the presence of preoperative midline shift and a higher level of Hb were associated with poor brain relaxation. Further investigations are necessary to confirm the relationship between Hb as well as other associations and brain swelling.
